# Primary Hepatic Lymphoma: A Challenging Diagnosis

**DOI:** 10.1155/2014/212598

**Published:** 2014-05-11

**Authors:** D. Myoteri, D. Dellaportas, E. Arkoumani, A. Marinis, A. Zizi-Sermpetzoglou

**Affiliations:** ^1^Pathology Department, Tzaneion General Hospital, 185 36 Piraeus, Greece; ^2^2nd Department of Surgery, University Hospital Aretaieion, 115 28 Athens, Greece; ^3^1st Department of Surgery, Tzaneion General Hospital, 185 36 Piraeus, Greece

## Abstract

*Introduction.* Primary hepatic lymphoma is an unusual malignancy and is very difficult to diagnose promptly. An intrigue case presenting with cholestatic jaundice is reviewed and main disease characteristics are further discussed. *Case Report.* A 70-year-old male presented with dull right upper quadrant abdominal pain and mild cholestatic jaundice. Initial evaluation revealed mildly elevated liver function tests and normal tumor markers, while imaging with an abdominal CT-scan showed multiple hypodense nodules in both liver lobes. First impression of metastatic deposits from gastrointestinal origin was not confirmed by endoscopic means. After CT-guided biopsy, primary diffuse large B-cells non-Hodgkin lymphoma was revealed. Appropriate chemotherapy improved patient's condition markedly. *Discussion.* Primary hepatic lymphoma is a rare form of extranodal lymphomas, accounting for less than 1% of all extranodal lymphomas in general. In order to define the condition as PHL, liver has to be the only site of lymphoma occurrence or to be involved in a major degree with minimal nonliver disease. Most PHLs are of B-cell origin with large cells as the main cell type.

## 1. Introduction

Primary hepatic lymphoma (PHL) is a very rare malignancy usually presenting with no specific symptoms leading to late diagnosis. Liver is thought to be an unwelcome environment for the development of malignant lymphoma, at least as a primary disease site [1]. We present an interesting case of primary diffuse large B-cells non-Hodgkin lymphoma (NHL) originating in the liver presenting as cholestatic jaundice. The clinical features, diagnostic measures, and management options are shortly discussed as well.

## 2. Case Presentation

**Figure 1:**
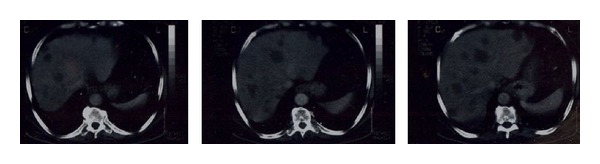
Abdominal CT-scan showing multiple hypodense nodules in both liver lobes.

**Figure 2:**
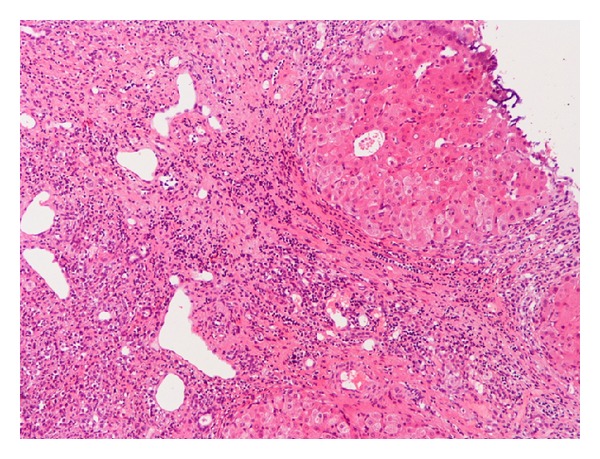
To the right, indicated uninvolved liver. To the left, atypical large lymphoid infiltrates at edges of portal tract, with disruption of hepatic parenchyma (H-E 40).

**Figure 3:**
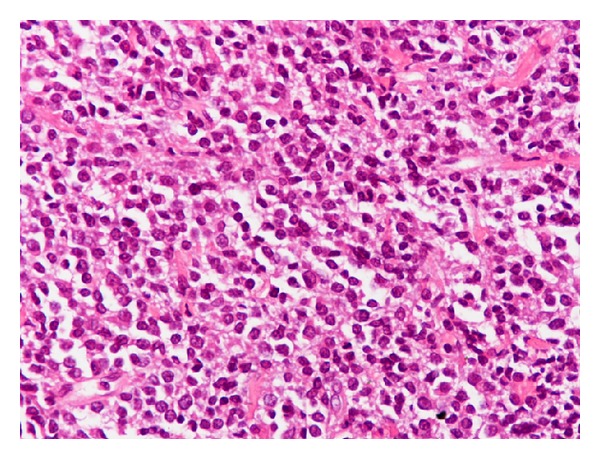
Uniform population of lymphoid cells of large size with many mitotic figures (H-E 400).

**Figure 4:**
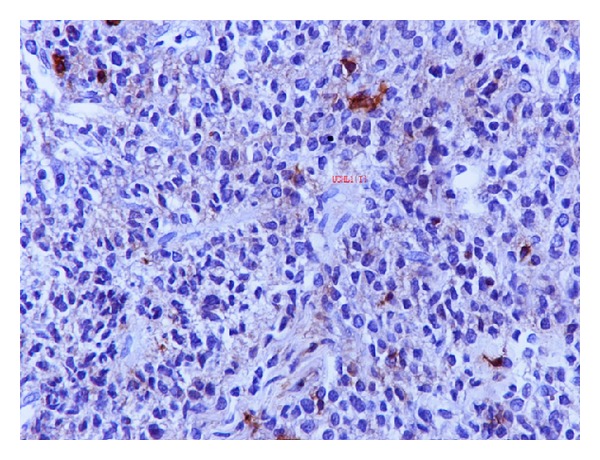
The neoplastic cells are negative for UCHL-1, a pan T-cell marker (anti-UCHL-1 200).

A 70-year-old man was admitted to our hospital with a dull ache on the right hypochondrium, myalgias, and mildly elevated total bilirubin levels (3.2 mg/dL). Patient did not mention any fever, night sweats, vomiting, chest pain, abdominal pain, diarrhea, blood in stools, or weight loss. His past medical history was significant for arterial hypertension and diabetes mellitus type II. On physical examination, mild epigastric tenderness was the only finding. Laboratory results included hematocrit 33%, hemoglobin levels 11.2 g/dL, and white cell count 9.8 × 10^9^/L, with 60% lymphocytes. Regarding liver function tests, transaminases levels were mildly elevated as well and lactic dehydrogenase (LDH) was 290 IU/L (normal range 100–190 IU/L). Initial differential diagnosis included virus hepatitis but serology for hepatic viruses was negative. An abdominal ultrasound scan showed multiple liver lesions as metastatic deposits. Carcinoembryonic antigen (CEA) and alpha-fetoprotein (AFP) as well as Ca 19–9 were within normal range. An abdominal computed tomography scan (CT-scan) followed which revealed multiple hypodense nodules in both liver lobes (Figure 1). Biliary tract was normal. Initial impression of metastatic deposits from gastrointestinal origin seemed reasonable. However, endoscopy of upper and lower gastrointestinal tract had no major findings. The patient was getting worse with further transaminase and bilirubin elevation, and hepatic encephalopathy developed as indicated by personality disorders and elevated ammonia levels. Finally, CT-guided biopsy of a nodule from the right liver lobe showed diffuse large B-cell lymphoma (Figures 2 and 3). The diagnosis was also confirmed with immunohistochemical studies (Figure 4). The patient was staged with bone-marrow biopsy and thorax CT-scan and no other foci of lymphoma were found in the body, so he received the diagnosis of multifocal PHL. He was promptly started on cyclophosphamide, doxorubicin, vincristine, and prednisone, with the addition of rituximab, known as the R-CHOP regimen, and improvement was markedly showed. The patient received 6 cycles of chemotherapy and had disease regression for one year and a half. Three years later, he developed recurrence and fulminant hepatic failure was the final event before multiorgan failure.

## 3. Discussion

PHL is an unusual disease and a rare form of extranodal lymphomas, accounting for less than 1% of all extranodal lymphomas in general. In order to define the condition as PHL, liver has to be the only site of lymphoma occurrence or to be involved in a major degree with minimal nonliver disease [2]. Etiologic factors implicated to be associated with PHL are Epstein-Barr virus (EBV) prior infection, hepatitis B and C (HBV, HCV), and cirrhosis [3]. Mostly HCV carriers are thought to be involved with PHL. Our patient however was negative for hepatitis viruses. A wide age range is reported from childhood to eighth life decade and men are affected twice as women [4]. Right upper quadrant pain or dullness, hepatomegaly, and palpable liver are the main presenting features. Jaundice and hepatic failure are also reported, while pleural effusion, thrombocytopenia, and fulminant hepatic failure are very unusual. Most of the time though diagnosis is an incidental finding, during investigations for nonspecific symptoms as nausea, mild abdominal discomfort, or early satiety, as in our case, hepatic infiltration may have a nodular or diffuse pattern with no actual prognostic value [5].

Most PHLs are diffuse large B-cell lymphomas (DLBCL), while T-cell PHLs are reported more infrequently. Other histologic subtypes of PHL include high-grade tumors (lymphoblastic and Burkett lymphoma, 17%), follicular lymphoma (4%), lymphoma of the mucosa-associated lymphoid tissue type, anaplastic large-cell lymphoma, mantle cell lymphoma, T-cell-rich B-cell lymphoma, and hepatosplenic T-cell lymphoma [6].

On laboratory data alkaline phosphatase (ALP) and lactic dehydrogenase (LDH) are elevated most of the time, while tumor markers as a-fetoprotein (AFP) and carcinoembryonic antigen (CEA) remain between normal ranges. Tumor markers help in differential diagnosis from hepatocellular carcinoma or metastatic disease.

Imaging modalities are helpful in defining a hypoechoic mass on ultrasonography (U/S) and a hypoattenuating lesion on computed tomography (CT) scan. Contrast injection leads to rim enhancement of the lesion. Usually magnetic resonance imaging (MRI) is not needed because biopsy of the lesion is the next step in diagnostic chain, but if done, it reveals a hypointense mass on T1-weighted images and hyperintense T2-weighted images [7]. Misdiagnosis happens and unnecessary resections are reported. Liver core biopsy under U/S or CT guidance is the next step as mentioned and leads to the correct diagnosis as long as liver is the only or main organ involved. Fine needle aspiration (FNA) of the lesion is usually inconclusive and that is the reason that tru-cut biopsy is the preferred pathway. Core biopsy specimens will also be sent for flow cytometry. That is why a bone-marrow biopsy and brain-thorax-abdominal CT-scan are performed.

Further and most sophisticated examinations have been developed in order to stratify DLBCL patients to those expressing a gene expression profile (GEP) of germinal center B-cells (GCBs), those who have a longer survival, and those with a GEP of activated B-cells (ABCs). DNA microarray technology is not practical for the analysis of routine patient samples. Immunohistochemistry methods are more easily applicable and CD10, BCL6, and MUM1 should be performed to determine whether it is germinal center type or activated B-cell type by the Ham criteria. In addition, a proliferative index by Ki-67 or MIB-1 staining is helpful, as well as paraffin FISH studies for BCL2, BCL6, and MYC rearrangements [8].

The mainstay of therapy for PHL patients is chemotherapy with CHOP regimen (cyclophosphamide, doxorubicin, vincristine, and prednisone), with the addition of rituximab (monoclonal antibody against CD-20), which prolongs survival with minimal toxicity, so the scheme is called R-CHOP [9]. This is a chemosensitive lesion, and clinical suspicion can help to avoid confusion with hepatocellular carcinoma or metastasis. Some oncologist would add radiotherapy in bulky lesions, but its efficacy is not proven.

Poor prognostic features are bulky disease with B-symptoms, elevated LDH and b2-microalbumin, high proliferation rate, and cirrhosis, with patients of older age and comorbid condition on worse favor as well [10].
